# Single-Cell Transcriptome Integration Analysis Reveals the Correlation Between Mesenchymal Stromal Cells and Fibroblasts

**DOI:** 10.3389/fgene.2022.798331

**Published:** 2022-03-07

**Authors:** Chuiqin Fan, Maochuan Liao, Lichun Xie, Liangping Huang, Siyu Lv, Siyu Cai, Xing Su, Yue Wang, Hongwu Wang, Manna Wang, Yulin Liu, Yu Wang, Huijie Guo, Hanhua Yang, Yufeng Liu, Tianyou Wang, Lian Ma

**Affiliations:** ^1^ Department of Pediatrics, The Second Affiliated Hospital of Shantou University Medical College, Shantou, China; ^2^ Department of Pediatrics, The Third Affiliated Hospital of Guangzhou Medical University (The Women and Children’s Medical Center of Guangzhou Medical University), Guangzhou, China; ^3^ Department of Hematology and Oncology, Shenzhen Children’s Hospital of China Medical University, Shenzhen, China; ^4^ Department of Pediatrics, The First Affiliated Hospital of Zhengzhou University, Zhengzhou, China; ^5^ Department of Hematology and Oncology, Beijing Children’s Hospital, Capital Medical University, Beijing, China

**Keywords:** fibroblast, mesenchymal stromal cells, integration analysis, pericytes, single-cell transcriptome sequencing

## Abstract

**Background:** Mesenchymal stromal cells (MSCs) and fibroblasts show similar morphology, surface marker expression, and proliferation, differentiation, and immunomodulatory capacities. These similarities not only blur their cell identities but also limit their application.

**Methods:** We performed single-cell transcriptome sequencing of the human umbilical cord and foreskin MSCs (HuMSCs and FSMSCs) and extracted the single-cell transcriptome data of the bone marrow and adipose MSCs (BMSCs and ADMSCs) from the Gene Expression Omnibus (GEO) database. Then, we performed quality control, batch effect correction, integration, and clustering analysis of the integrated single-cell transcriptome data from the HuMSCs, FMSCs, BMSCs, and ADMSCs. The cell subsets were annotated based on the surface marker phenotypes for the MSCs (*CD105* ^*+*^, *CD90* ^+^, *CD73* ^+^, *CD45* ^−^, *CD34* ^−^, *CD19* ^−^, *HLA-DRA* ^−^, and *CD11b* ^−^), fibroblasts (*VIM* ^+^, *PECAM1* ^−^, *CD34* ^−^, *CD45* ^−^, *EPCAM* ^−^, and *MYH11* ^−^), and pericytes (*CD146* ^+^, *PDGFRB* ^+^, *PECAM1* ^−^, *CD34* ^−^, and *CD45* ^−^). The expression levels of common fibroblast markers (*ACTA2*, *FAP*, *PDGFRA*, *PDGFRB*, *S100A4*, *FN1*, *COL1A1*, *POSTN*, *DCN*, *COL1A2*, *FBLN2*, *COL1A2*, *DES*, and *CDH11*) were also analyzed in all cell subsets. Finally, the gene expression profiles, differentiation status, and the enrichment status of various gene sets and regulons were compared between the cell subsets.

**Results:** We demonstrated 15 distinct cell subsets in the integrated single-cell transcriptome sequencing data. Surface marker annotation demonstrated the MSC phenotype in 12 of the 15 cell subsets. C10 and C14 subsets demonstrated both the MSC and pericyte phenotypes. All 15 cell subsets demonstrated the fibroblast phenotype. C8, C12, and C13 subsets exclusively demonstrated the fibroblast phenotype. We identified 3,275 differentially expressed genes, 305 enriched gene sets, and 34 enriched regulons between the 15 cell subsets. The cell subsets that exclusively demonstrated the fibroblast phenotype represented less primitive and more differentiated cell types.

**Conclusion:** Cell subsets with the MSC phenotype also demonstrated the fibroblast phenotype, but cell subsets with the fibroblast phenotype did not necessarily demonstrate the MSC phenotype, suggesting that MSCs represented a subclass of fibroblasts. We also demonstrated that the MSCs and fibroblasts represented highly heterogeneous populations with distinct cell subsets, which could be identified based on the differentially enriched gene sets and regulons that specify proliferating, differentiating, metabolic, and/or immunomodulatory functions.

## Introduction

Mesenchymal stromal cells (MSCs) are multipotent adult stromal cells that are used for tissue repair (Maqsood et al., 2020) and immunomodulation (Mo et al., 2016). MSCs have been applied to the treatment of a variety of diseases, such as steroid-refractory graft-versus-host disease (Le Blanc et al., 2004), diabetes (Bhansali et al., 2017), multiple sclerosis (Cohen et al., 2018), cardiac ischemic injury (Qayyum et al., 2017), systemic lupus erythematosus (Wang et al., 2014), inflammatory bowel disease (Dhere et al., 2016), osteoarthritis (Lamo-Espinosa et al., 2018), and sepsis (Simonson et al., 2015). MSCs were first isolated from the bone marrow (Friedenstein et al., 1974) and subsequently from other sources, such as the umbilical cord Wharton’s jelly (Mitchell et al., 2003), adipose tissue (Zuk et al., 2001), foreskin (Najar et al., 2016), synovial fluid (De Bari et al., 2001), dental pulp (Gronthos et al., 2000), and endometrium (Schwab et al., 2008). MSCs derived from different tissues consist of heterogenous cellular populations with distinct biological properties (Mattar and Bieback, 2015). Fibroblasts are the most common type of stromal cells found abundantly in the connective tissues; they secrete proteins that constitute the extracellular matrix and play an essential role in wound repair, tissue development, and fibrosis (Muhl et al., 2020). Fibroblasts have also been isolated from various tissues, such as the cornea, skin, adipose tissue, heart, skeletal muscle, intestine, and bladder (Chang et al., 2002; Muhl et al., 2020).

MSCs are isolated based on the International Association for Cell Therapy (ISCT) criteria (Dominici et al., 2006), which includes (1) adherent growth in plastic Petri dishes; (2) high expression of surface markers such as CD73, CD90, and CD105 and low expression or absence of HLA-DR, CD11b or CD14, CD19 or CD79a, CD34, and CD45 expression; and (3) the ability to differentiate into osteoblasts, chondrocytes, and adipocytes *in vitro*. The fibroblasts demonstrate adherent growth in plastic Petri dishes with a spindle or wide flat shape (Alt et al., 2011). Fibroblasts also express CD73, CD90, and CD105 and lack the expression of HLA-DR, CD11b or CD14, CD19 or CD79a, CD34, and CD45 (Chen et al., 2007; Haniffa et al., 2007; Alt et al., 2011; Blasi et al., 2011; Soundararajan and Kannan, 2018). Moreover, fibroblasts demonstrate the potential to differentiate into osteoblasts (Chen et al., 2007; Blasi et al., 2011), chondroblasts (Chen et al., 2007; Alt et al., 2011), and adipocytes (Chen et al., 2007; Blasi et al., 2011) under specialized growth conditions. Therefore, fibroblasts show significant similarity with the MSCs based on the ISCT criteria. Furthermore, both MSCs and fibroblasts exert immunosuppressive effects through cell–cell interactions and paracrine mechanisms (Soundararajan and Kannan, 2018; Ugurlu and Karaoz, 2020). MSCs and fibroblasts also show similarities in cell morphology (Alt et al., 2011), multipotent differentiation and replicative ability (Alt et al., 2011), gene expression profile (Bae et al., 2009), and immunosuppressive functions (Haniffa et al., 2007). Besides, MSCs and fibroblasts also show similar plasticity and stemness as they can be induced to differentiate into hepatocytes, cardiomyoblasts, islet cells, muscle cells, and germ cells (Chen et al., 2015; Maldonado et al., 2017; Zhang et al., 2019; Ugurlu and Karaoz, 2020) and can be reprogrammed into induced pluripotent stem cells (iPSCs) (Takahashi and Yamanaka, 2006; Buccini et al., 2012). It is also postulated that the differences in methylation patterns between the MSCs and fibroblasts (de Almeida et al., 2016) are related to the donor’s age and long-term culturing (Koch et al., 2011). Furthermore, fibroblast surface markers have been reported in senescent MSCs (Soundararajan and Kannan, 2018). This suggests that both cell types are essentially the same type of cells in different life cycle stages; that is, fibroblasts may represent aging MSCs, and MSCs may represent naive fibroblasts (Soundararajan and Kannan, 2018). In contrast to this view, some studies suggested that fibroblasts do not represent aging MSCs and were differentiated from the MSCs under certain conditions (Mishra et al., 2009; Chan et al., 2019). Regardless of these conflicting views, it is generally accepted that fibroblasts are closely related to MSCs. However, it is important to clearly distinguish the MSCs from the fibroblasts because MSCs are used for several clinical applications. Therefore, in this study, we performed comprehensive bioinformatics analysis of integrated single-cell transcriptome sequencing data from the human umbilical cord mesenchymal stromal cells (HuMSCs), foreskin mesenchymal stromal cells (FSMSCs), bone marrow mesenchymal stromal cells (BMSCs), and adipose mesenchymal stromal cells (ADMSCs) to identify distinct cell subsets that show similarities or differences in gene expression patterns, biological functions, and transcriptional regulatory networks between the MSCs and fibroblasts.

## Methods

### Data Collection and Ethical Approval

The single-cell transcriptome data of the BMSCs and ADMSCs were extracted from the GEO database [BMSCs: GSE115149 (Jitschin et al., 2019) and GSE162692 (Ruoss et al., 2021); ADMSCs: SRP148833 (Liu et al., 2019)]. These data were available for the public and did not require ethical approval. Furthermore, the collection of umbilical cords and foreskin tissues was approved by The Second Affiliated Hospital of Shantou University Medical College of China (Institutional Review Board approval numbers: 2020-11 and 2021-89). The white connective tissues of the umbilical cord and the dermal tissues of the foreskin were cut into small pieces and cultured with Dulbecco’s Modified Eagle Media/Nutrient Mixture F-12 (Gibco, United States) containing 10% fetal bovine serum (Gibco, United States) to generate the primary HuMSCs and FSMSCs, respectively. MSCs from the third passage were subjected to single-cell transcriptome sequencing using the 10× Genomics Chromium sequencing platform. The sequencing data of the HuMSCs and FSMSCs were processed with the 10× Genomics Cell Ranger software (version 3.1.0) (Zheng et al., 2017) and registered at the Mendeley database (https://data.mendeley.com/datasets/f4b2ykfv56/1).

### Quality Control and Integration

The gene expression data of the BMSCs from the control and unprocessed groups were obtained from the GSE115149 (platform: 10× Genomics Chromium, passage: 3) and GSE162692 (platform: 10 × Genomics Chromium, passage: unknown) datasets. The gene expression data of the ADMSCs were obtained from the SRP148833 dataset (platform: 10 × Genomics Chromium, passage: 3) using the 10× Genomics Cell Ranger software (Zheng et al., 2017). Then, the Seurat objects for the BMSCs, ADMSCs, HuMSCs, and FSMSCs were created using the Seurat R package (version 4.0.0) (Hao et al., 2020). The quality control screening included retaining high-quality cells after excluding data for cells that contained less than 200 genes or more than 20,000 genes and/or greater than 20% mitochondrial genes. Finally, we merged all the filtered Seurat objects from the high-quality cells.

### Data Processing

The merged data were normalized and standardized to correct the sequencing depth of each cell. The principal component analysis (PCA) was performed based on the 2,000 genes with high variability to acquire the first 50 principal components (PCs), which were then subjected to batch effect correction using the R package harmony (version 1.0) (Korsunsky et al., 2019). The first 50 harmony dimensions were used for *t*-distributed stochastic neighbor embedding (TSNE) with a resolution of 0.5. The cluster tree for the cell subsets of cells was constructed using the BuildClusterTree function of Seurat, and the cell subsets were annotated.

### Analysis of the Cell Subsets

The expression levels of the marker genes representing the MSC, fibroblast, and pericyte phenotypes were calculated for the different cell subsets using the Findallmarker function of the R package Seurat with an adjusted *p*-value <0.05 and an absolute value of log2 fold change (Log2FC) ≥1. Then, gene set enrichment analysis was performed to evaluate the potential biological functions of the cellular subsets by calculating the enrichment scores and *p*-value of the hallmark gene sets in different cell subsets using the R package msigdbr (version 7.2.1) (Dolgalev, 2020) and singleseqgset (version 0.1.2.9000) (Cillo, 2021). The gene sets with a *p*-value <0.5 were regarded as statistically significant. Finally, the differentiation degree of each cell subset was calculated using the R package CytoTRACE (version 1.8.0) (Gulati et al., 2020).

### Analysis of Gene Regulatory Networks

The co-expression modules of different transcription factors and their target genes were analyzed from the gene expression data using the python package pySCENIC (version 0.10.3) (Aibar et al., 2017). Then, the significant motifs in the co-expression module were analyzed by the motif enrichment analysis after deleting the target genes with low scores. The target genes and their corresponding transcription factors in the co-expression module were considered as a regulon. The regulon activity score (RAS) of each regulon in a cell was calculated. Then, the RAS threshold of each regulon was estimated. If RAS was greater than the threshold, the regulon in the cell was considered as activated. Otherwise, the regulon was considered as silent. The RAS matrix was transformed into a binary matrix based on the “0/1” scoring according to the threshold value to eliminate technical bias and identify the differences. Finally, the regulon-specific scores (RSS) of different regulons in the cellular subsets were calculated using the R package philentropy (version 0.4.0) (HG, 2018). The first five regulons (highly expressed) in different cell subsets were then filtered and analyzed.

## Results

### Quality Control

Seurat objects were created for BMSCs, ADMSCs, HuMSCs, and FSMSCs, and quality control parameters were set to retain cells with expression readouts between 200 and 2,000 genes and ≤20% mitochondrial genes per cell. Subsequently, we identified 5,180 high-quality BMSCs (median UMI: 12,340; median genes: 3,058), 29,178 high-quality ADMSCs (median UMI: 40,208; median genes: 5,602), 13,386 high-quality HuMSCs (median UMI: 14,854; median genes: 3,797), and 7,432 high-quality FSMSCs (median UMI: 33,022; median genes: 5,463).

### Integration Analysis

**FIGURE 1:**
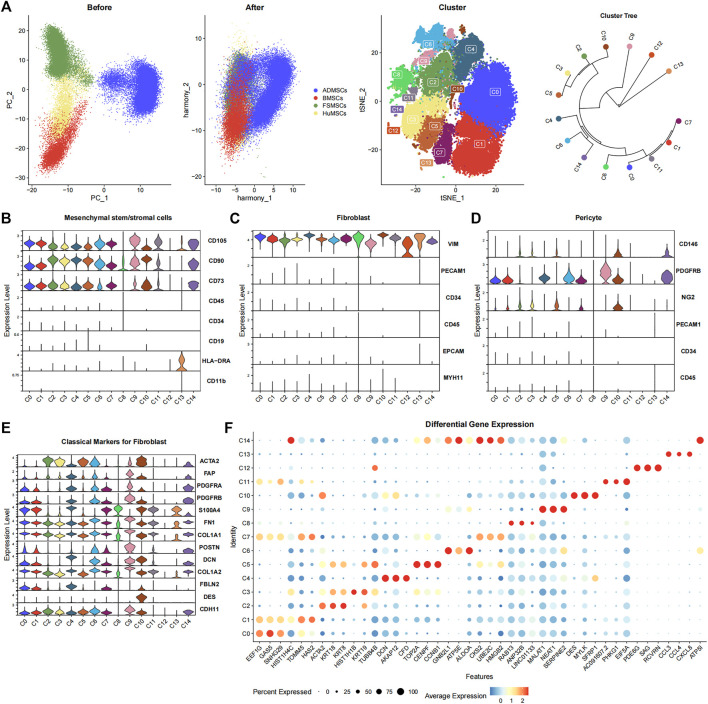
Characterization of the 15 cell subsets derived from ADMSCs, BMSCs, FSMSCs, and HuMSCs using various bioinformatics analysis methods. **(A)** The PCA plots show the distribution of ADMSCs (blue), BMSCs (red), FSMSCs (green), and HuMSCs (yellow), which are derived from different tissues before and after correcting the batch effects. The TSNE plot shows distribution of the 15 cell subsets (C0–C14). The clustering tree shows similarity between the 15 cell subsets. **(B**–**D)** The violin plot shows the expression status of **(** **B** **)** MSC-related markers, **(** **C** **)** fibroblast-related markers, and **(** **D** **)** pericyte-related markers in the 15 different cell subsets. **(** **E** **)** The violin plot shows the expression of classical fibroblast markers in the 15 different cell subsets. **(** **F** **)** The bubble plot shows the differential expression of the top three highly expressed genes in each cellular subset among all the 15 cellular subsets. The size of the bubble is inversely proportional to the *p*-value; that is, the larger the bubble, the smaller the *p*-value. The expression level is color coded, with dark red representing high expression and blue representing low expression.

The merged data were normalized and standardized to correct the sequencing depth of each cell. We identified the first 50 PCs using PCA based on 2,000 genes with high variance. Then, the 50 PCs were used to correct the batch effect, and the first 50 harmony dimensions were identified. The reduction plot between PCA dimensionality and harmony dimensionality showed a significant overlap between MSCs derived from the same tissue and partial overlap between MSCs derived from different tissues (Figure 1A). This suggested an effective correction of the batch effects from different datasets and resulted in identification of biological differences between the MSCs derived from different tissues. The first 50 harmony dimensions were also used for TSNE plots with a clustering resolution of 0.5, and 15 major cell subsets were identified (Figure 1A). The cluster tree was constructed by the BuildClusterTree function of the R package Seurat to analyze the correlations between the 15 cell subsets, and the tree plot was displayed using the R package ggtree (version 2.4.1) (Yu, 2020) (Figure 1A).

### Biological Annotations

The 15 cell subsets were annotated based on the surface marker expression profiles of MSCs (*CD105* ^*+*^, *CD90* ^*+*^, *CD73* ^*+*^, *CD45* ^*−*^, *CD34* ^*−*^, *CD19* ^*−*^, *HLA-DRA* ^−^, and *CD11b* ^−^) (Dominici et al., 2006) (Figure 1B). The expression of *CD105*, *CD90*, and *CD73* was significantly high, and the expression of *CD45*, *CD34*, *CD19*, *HLA-DRA*, and *CD11b* was significantly low or absent in 12 cell subsets, namely, C0–C7, C9–C11, and C14. This suggested that these 12 cell subsets were the MSCs. C8 and C13 subsets showed lower expression of *CD90* and *CD105*, respectively. Therefore, C8 and C13 did not show the classical MSC phenotype. The 12 cell subsets expressed *VIM* but did not express *PECAM1*, *CD34*, *CD45*, *EPCAM*, and *MYH11* (Figure 1C). This suggested that all the 12 cell subsets also showed the fibroblast phenotype. Furthermore, we observed the expression of fibroblast markers such as *ACTA2* (Muhl et al., 2020), *FAP* (Sunami et al., 2020), *PDGFRA* (Muhl et al., 2020), *PDGFRB* (Muhl et al., 2020), *S100A4* (Meng et al., 2020), *FN1* (Meng et al., 2020), *COL1A1* (Meng et al., 2020), *POSTN* (Meng et al., 2020), *DCN* (Guerrero-Juarez et al., 2019), *COL1A2* (Meng et al., 2020), *FBLN2* (Guerrero-Juarez et al., 2019), *DES* (Sunami et al., 2020), and *CDH11* (Tarbit et al., 2019) in one or more cellular subsets (Figure 1E). This demonstrated that the cell subsets could not be distinguished using a single classical fibroblast marker. The pericyte surface markers such as *CD146* ^*+*^, *PDGFRB* ^*+*^, *PECAM1* ^−^, *CD34* ^−^, and *CD45* ^−^ (Crisan et al., 2008; Crisan et al., 2012) were expressed in some cell subsets. C10 and C14 highly expressed *CD146* and *PDGFRB* but did not express *PECAM1*, *CD34*, and *CD45*, suggesting that both of them met the pericyte phenotype (Figure 1D). This suggested that some cell subsets represented overlapping pericyte and MSC phenotypes.

### Biological Function Analysis

The Findallmarker function of R package Seurat filtered out 3,275 statistically significant genes using the adjusted *p*-value < 0.05 and the absolute value of Log2FC ≥ 1 (Supplementary Table S1). This included 80, 65, 91, 127, 146, 139, 215, 39, 35, 554, 80, 44, 931, 502, and 227 genes in the C0, C1, C2, C3, C4, C5, C6, C7, C8, C9, C10, C11, C12, C13, and C14 subsets, respectively. The bubble plot shows the top three highly expressed differential genes from each cell subset (Figure 1F).

**FIGURE 2:**
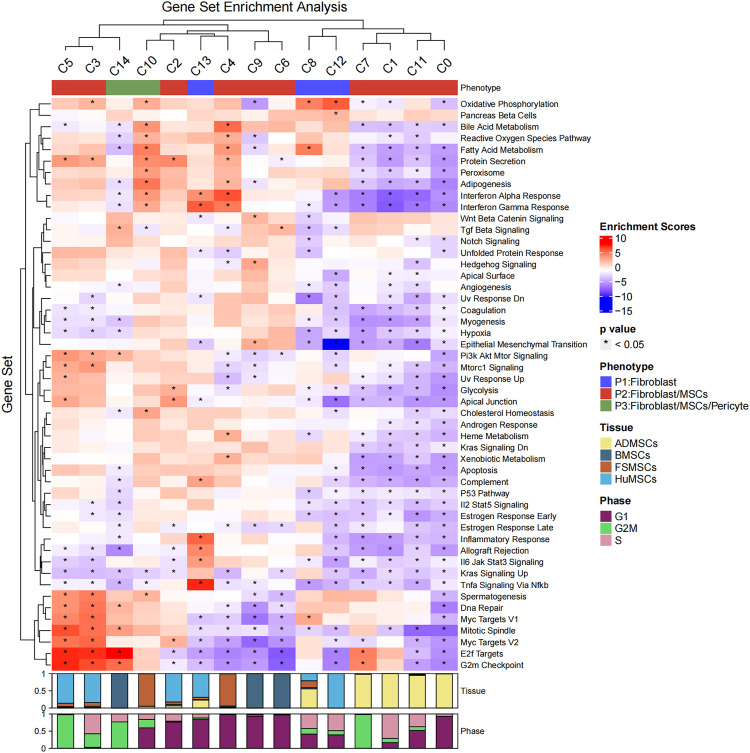
Hallmark gene set enrichment analysis of the 15 cell subsets. The heatmap plot shows the enrichment scores and *p*-values of different hallmark gene sets among the 15 different cellular subsets. The color of the grids denotes the enrichment scores of the gene set, with red representing high enrichment and blue representing low enrichment. The bar graphs (top) represent different molecular phenotypes (fibroblasts, MSCs, and/or pericytes) represented by the 15 different cell subsets and is shown with different colors. The asterisk indicates that the *p*-value was less than 0.05. The bar charts (below) represent the cell cycle phases of different proportions of cells in the 15 cell subsets.

Then, the hallmark gene set enrichment analysis was performed using the R package msigdbr and singleseqgset. The enrichment scores and *p*-values of all hallmark gene set were compared between the 15 cell subsets. We identified 305 differentially expressed gene sets using an adjusted *p*-value of less than 0.05 as the cutoff (Figure 2; Supplementary Table S2). The C0, C1, C2, C3, C4, C5, C6, C7, C8, C9, C10, C11, C12, C13, and C14 cell subsets showed significant differences in the expression of genes belonging to 41, 33, 8, 41, 29, 13, 18, 3, 16, 15, 16, 10, 29, 16, and 17 gene sets, respectively. The biological functions of different cell subsets are shown in Figure 2. The gene set analysis showed that the C3, C4, C5, C7, C10, and C14 cell subsets demonstrated both MSC and fibroblast phenotypes. Proliferation-related gene sets such as “E2F Target genes” and “G2M Checkpoint genes” were enriched in the C3, C5, C7, and C14 subsets. The metabolism-related gene sets belonging to “Oxidative Phosphorylation,” “Bile Acid Metabolism,” “Reactive Oxygen Species Pathway,” “Fatty Acid Metabolism,” “Protein Secretion,” “Hypoxia,” “Glycolysis,” and “Heme Metabolism” were downregulated in the C0, C2, C1, and C11 subsets. The “Protein Secretion” gene set was upregulated in the C3 and C5 subsets. Immune-related gene sets belonging to “Interferon Alpha Response” and “Interferon Gamma Response” were enriched in the C4, C10, and C13 subsets. The C13 subset demonstrated only fibroblast phenotype with a high expression of HLA-DRA and absence of *CD80* and *CD86* expression (Supplementary Figure S1).

**FIGURE 3:**
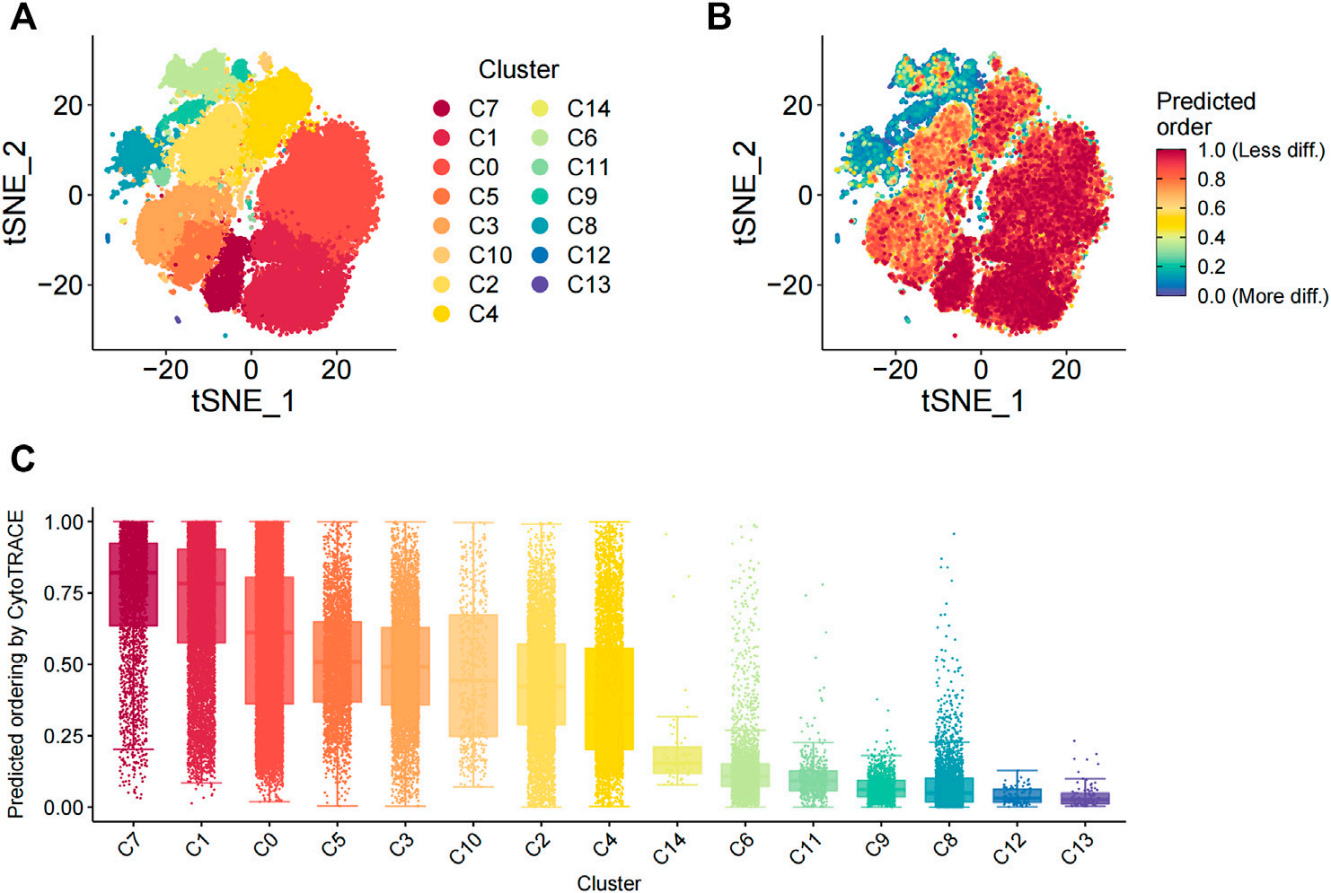
Evaluation of the differentiation status of cells in the 15 cellular subsets. **(** **A** **)** TSNE_plot shows the distribution of the 15 different cell subsets with different color codes in the low-dimensional space. **(** **B** **)** TSNE plot shows the distribution of the 15 different cell subsets based on the degree of differentiation. The degree of differentiation is denoted by the color code, with red representing low differentiation status and blue denoting high differentiation status. **(** **C** **)** The box plot shows the differentiation status of all cells in the 15 different cell subsets. The cells are ordered from low to high degrees of differentiation.

The differentiation status of the cell subsets was evaluated using the R package CytoTRACE. The batch effect correction was performed using the iCytoTRACE function. The CytoTRACE scores of different cell subsets are shown in the TSNE plots (Figures 3A,B) and box plots (Figure 3C). The differentiation status was considered lower when the CytoTRACE score was closer to 1.0. C8, C12, and C13 cell subsets showed CytoTRACE scores closer to 0.0 and were considered to belong to a higher differentiation state. C8, C12, and C13 demonstrated only the fibroblast phenotype and did not show characteristics of the MSC phenotype. This suggested that MSC characteristics are lost as they undergo differentiation. Furthermore, the differentiation status of the C14 and C10 cell subsets was not low despite showing the pericyte phenotype.

### Gene Regulatory Networks Analysis

**FIGURE 4:**
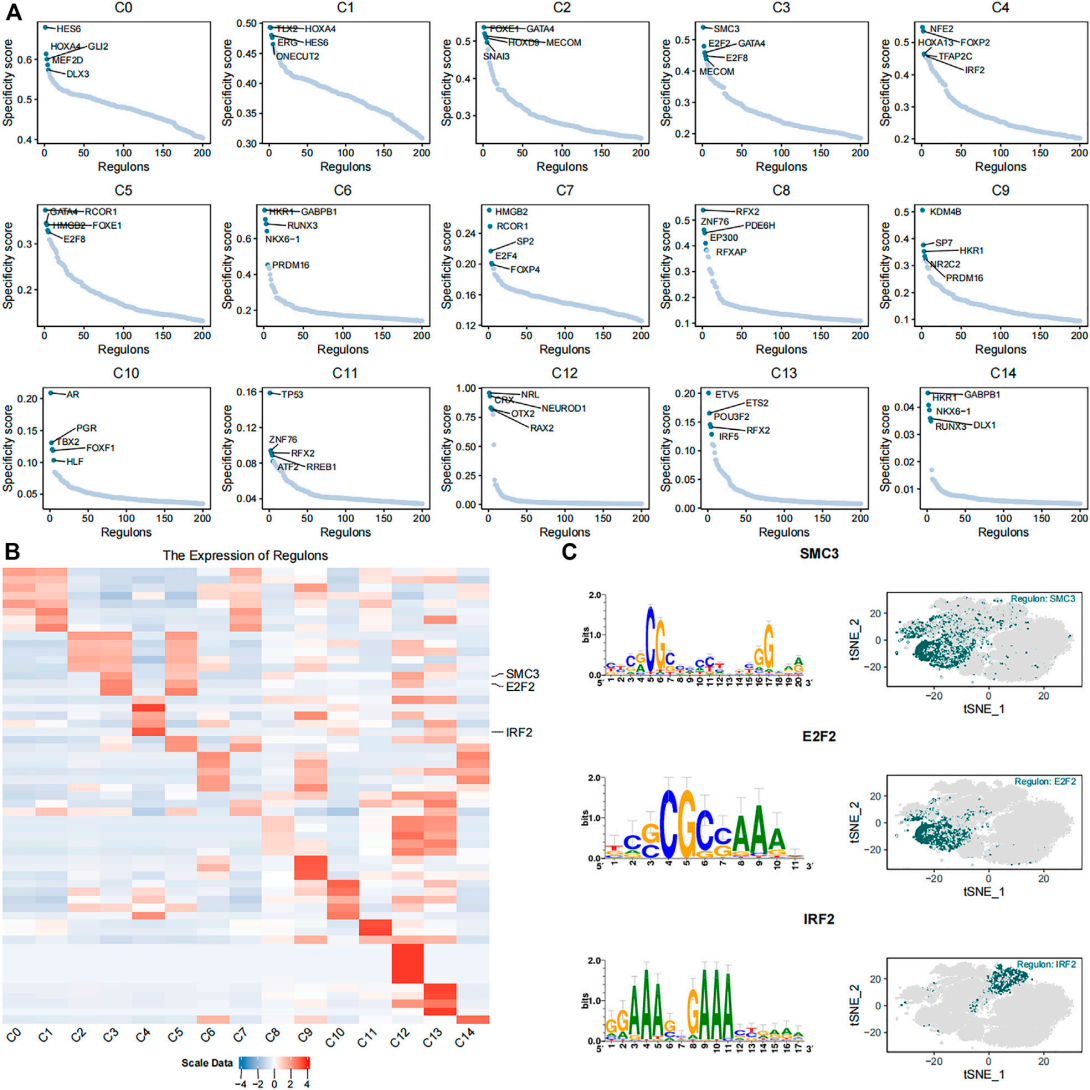
Identification of key gene regulatory networks in the 15 cell subsets. **(** **A** **)** The scatter plots show expression of the top five highly expressed regulons in each of the 15 cell subsets. **(** **B** **)** The heatmap plot shows expression levels of the top five highly expressed regulons in the 15 different cell subsets. *SMC3*, *E2F2*, and *IRF2* regulons are labeled. **(** **C** **)** The plots show the putative binding motifs of *SMC3*, *E2F2*, and *IRF2*, as well as their corresponding expression levels in different cells among the 15 cell subsets.

We identified 384 regulons by integrating the single-cell transcriptomic data using the pySCENIC python package. Each regulon contained a transcription factor, a significant motif, and the corresponding target genes (Supplementary Table S3). Regulon activity scores (RASs) of different regulons in each cell were evaluated. The RAS threshold of each regulon was also calculated separately. The regulon was considered as activated when the RAS value of the regulon was greater than its RAS threshold. Otherwise, the regulon was considered as silent. The RAS matrix was transformed into a binary matrix to highlight the differences between different cell subsets and eliminate the technical bias based on the threshold value. Furthermore, the regulon specificity score (RSS) of each regulon was calculated in different subsets using the philentropy R package (Figure 4A). The heatmap shows the top five regulons in each cell subset based on the regulon specificity scores (Figure 4B). The C3 subset showed high expression of DNA replication regulators such as *SMC3* (Gregson et al., 2001) and *E2F2* (Lukas et al., 1996; Delgado et al., 2011) (Figure 4C). The cells in the C3 subtype were mainly derived from the umbilical cord tissue. The C4 subset showed high expression of *IRF2* (Figure 4C), which exerts anti-inflammatory effects through inhibition of pro-inflammatory factors that are induced by lipopolysaccharide (Cui et al., 2018). The cells in the C4 subset were mainly derived from the foreskin tissue.

## Discussion

Traditional bulk transcriptome sequencing detects differences in gene expression between groups of cells, whereas single-cell transcriptome sequencing detects differences in gene expression between individual cells (Yan et al., 2021). Therefore, single-cell transcriptome sequencing technique demonstrates the composition of cells with differential functions in heterogeneous cell groups such as tumor cells, MSCs, and fibroblasts. In this study, we comprehensively analyzed the integrated single-cell transcriptome data of MSCs derived from four different tissues, namely, BMSCs, ADMSCs, HuMSCs, and FSMSCs and identified 15 cell subsets. Then, we performed cluster annotation of these 15 cell subsets based on the surface expression characteristics of MSCs (*CD105* ^*+*^, *CD90* ^+^, *CD73* ^+^, *CD45* ^−^, *CD34* ^−^, *CD19* ^−^, *HLA-DRA* ^−^, and *CD11b* ^−^), fibroblasts (*VIM* ^+^, *PECAM1* ^−^, *CD34* ^−^, *CD45* ^−^, *EPCAM* ^−^, and *MYH11* ^−^), and pericytes (*CD146* ^+^, *PDGFRB* ^+^, *PECAM1* ^−^, *CD34* ^−^, and *CD45* ^−^).

We identified MSCs based on the ISCT criteria, which is commonly used in most studies (Dominici et al., 2006). However, CD34 expression is observed sometimes in the early passages of freshly isolated ADMSCs (Baer et al., 2013). This suggested that the CD34 expression was highly variable between the donors. ADMSCs from some donors were CD34^+^, whereas those from other donors were CD34^−^. Moreover, CD34 expression decreased with the increasing number of passages of ADMSC cultures *in vitro*. Another single-cell RNA-sequencing study showed that 0.008% of cultured human ADMSCs were CD34^+^ (Liu et al., 2019). Furthermore, one review hypothesized that *in vitro* cultured MSCs were CD34^−^, whereas tissue-resident MSCs were CD34^+^ (Lin et al., 2012). The minimal ISCT criteria suggest that CD34 positivity in the MSCs must be ≤2% based on the flow cytometry analysis. This suggests that the proportion of CD34^+^ cells in the *in vitro* cultured MSCs is low. In our study, MSCs of all the datasets were cultured *in vitro*. Therefore, CD34 was used as a negative marker for determining the MSC phenotype.

Standardized identification criteria do not exist for fibroblasts despite being isolated, cultured, and characterized prior to the MSCs. Few reports suggest that fibroblasts can be identified based on vimentin (VIM) expression (Chang et al., 2002; Tarbit et al., 2019). VIM is the main structural component of the intermediate filaments in cells and is responsible for biological functions such as cell contraction, migration, and proliferation. However, *VIM* is also expressed in the endothelial cells, epithelial cells, and immune cells. Therefore, markers for endothelial cells (*PECAM1*), hematopoietic cells (*CD34*), immune cells (*CD45*), epithelial cells (*EPCAM*), and smooth muscle cells (*MYH11*) need to be tested alongside VIM to classify fibroblasts. A report also showed that fibroblasts express HLA-DR (Olsson et al., 1994). However, a comparative study showed that unstimulated fibroblasts and MSCs were both negative for HLA-DR (Denu et al., 2016). This suggested that HLA-DR expression was heterogeneous in the fibroblasts and was not necessary for identifying fibroblasts.

In our study, cluster annotation demonstrated that all the 15 cell subsets expressed fibroblast-related markers. However, only 12 cell subsets (C0, C1, C2, C3, C4, C5, C6, C7, C9, C10, C11, and C14) expressed MSC-specific markers. The remaining three cell subsets, namely, C8, C12, and C13, exclusively expressed fibroblast-specific markers. This suggested that MSCs may be derived from the fibroblasts or may represent a subclass of fibroblasts. However, MSCs are not equivalent to the fibroblasts because our single-cell transcriptome analysis demonstrates subtle differences in gene expression between the MSCs and the fibroblasts. Furthermore, the expression of classic fibroblast markers (ACTA2, FAP, PDGFRA, PDGFRB, S100A4, FN1, COL1A1, POSTN, DCN, COL1A2, FBLN2, COL1A2, DES, and CDH11) was not uniform among the 15 cell subsets. Hence, a single classic fibroblast marker was not sufficient to sort out all the cell subsets that show the fibroblast phenotype. Therefore, multiple classic fibroblast-specific markers are required to sort out all the fibroblast subsets because individual markers may not be expressed in some fibroblast subsets.

The C10 and C14 cell subsets with the MSC phenotype also demonstrated the pericyte phenotype. The expression of *NG2* was positive in C10 and negative in C14. This was consistent with a previous study that reported NG2 expression in only few pericyte subsets (Crisan et al., 2012). *NG2* is mainly distributed on the surface of vascular pericytes, MSCs, hematopoietic stem cells, and other pluripotent stem cells, which participates in angiogenesis process and regulates stem cell differentiation, stemness maintenance, and self-renewal. Our study demonstrated some overlap between the pericytes and MSCs.

We also analyzed the differences in the status of differentiation between the 15 cell subsets. C8, C12, and C13 subsets that demonstrated only the fibroblast phenotype were more differentiated than the other 12 cell subsets, which demonstrated both fibroblast and MSC phenotypes. This implied that MSCs represented a more primitive cellular stage that gradually disappeared as the cells underwent differentiation. Furthermore, C10 and C14 cell subsets with the pericyte phenotype did not represent the least differentiated cells. This finding was not consistent with a previous study, which suggested that MSCs were derived from the pericytes (Crisan et al., 2008). This suggested that MSCs may be derived from multiple lineages.

We then analyzed the differences in the biological functions between the 15 cell subsets. Our results showed that although the C3, C4, C5, C7, C10, and C14 subsets demonstrated both MSC and fibroblast phenotypes, the proliferation-related gene sets such as “E2F Targets” and “G2M Checkpoint” were enriched only in the C3, C5, C7, and C14 subsets. This suggested that cells in the C3, C5, C7, and C14 subsets were proliferating and in the G2M or S phase of the cell cycle. The C5 and C3, C14, and C7 subsets were mainly derived from the umbilical cord, bone marrow, and adipose tissues, respectively. Our data suggested that the proliferation activity of the HuMSCs, BMSCs, and ADMSCs was significantly higher than the FSMSCs. The C2 subset was mainly derived from the umbilical cord; C9 and C6 subsets were mainly derived from the bone marrow; the C10 subset was mainly derived from the adipose tissue. However, proliferation-related “E2F Target” and “G2M Checkpoint” gene sets were not enriched in these four cell subsets. This suggested that MSCs and fibroblasts consisted of heterogeneous cell populations with different biological functions. These findings were consistent with previous reports (Dunn et al., 2021; Plikus et al., 2021; Wruck et al., 2021; Zou et al., 2021). In the C0, C1, C2, and C11 subsets, metabolism-related genes belonging to “Oxidative Phosphorylation,” “Bile Acid Metabolism,” “Reactive Oxygen Species Pathway,” “Fatty Acid Metabolism,” “Protein Secretion,” “Hypoxia,” “Glycolysis,” and “Heme Metabolism” gene sets were downregulated. These four cell subsets were mainly derived from the adipose tissues. This suggested that ADMSCs were metabolically inactive. The “Protein Secretion” gene set was upregulated in the C3 and C5 subsets, which were mainly derived from the umbilical cord. This suggested that the exocrine functions were activated in the HuMSCs. The immune-related “Interferon Alpha Response” and “Interferon Gamma Response” gene sets were enriched in the C4 and C10 subsets, which are derived from the foreskin tissues. The interferon alpha and interferon gamma response genes are essential components of the immune response to viral infections. This suggested that the foreskin-derived MSCs may play an important role in response to inflammation (González-Navajas et al., 2012). Furthermore, foreskin is a source of immunotherapeutic MSCs (Najar et al., 2016). MSCs derived from the foreskin tissue significantly promote the increase of the proportion of Th17 cells (Najar et al., 2021). Moreover, in our unpublished manuscript (Supplementary Material), we demonstrated through *in vitro* experiments that the immunomodulatory capacity of the FSMSCs was significantly higher than the HuMSCs. This suggested the potential clinical significance of FSMSCs in treating diseases related to immune regulation. The C13 cell subset that demonstrated only the fibroblast phenotype was enriched with both “Interferon Alpha Response” and “Interferon Gamma Response” gene sets. Furthermore, the C13 subset showed high expression of *HLA-DRA*, thereby indicating stronger immunogenicity. However, the expression of co-stimulatory factors, *CD80* and *CD86*, was not detected in the C13 subtype. This suggested that the *HLA-DRA* ^*+*^ phenotype of the fibroblasts does not directly correlate with immunogenicity. *HLA-DRA* seemed to indicate that C13 might possess stronger immunogenicity.

The plasticity of MSCs and fibroblasts may be a likely source of heterogeneity (Lindsay and Barnett., 2021; Plikus et al., 2021; Rauch and Mandrup, 2021). The gene regulatory networks play a vital role in maintaining the plasticity of the MSCs and fibroblasts. The C3 subset showed high expression of the cell-cycle-related regulons, *SMC3* and *E2F2*, whereas anti-inflammatory-related regulon *IRF2* was highly expressed in the C4 cell subset. This suggested that cells in the C3 subset were proliferative, whereas cells in the C4 subset may play an essential role in the inflammatory responses. We speculate that the differences in the transcriptional regulatory networks may be responsible for the diversity of biological functions observed in distinct cell subsets derived from the same source or within cells demonstrating either MSC or fibroblast phenotypes.

Both MSCs and fibroblasts are multipotent stromal cell populations that can be induced to differentiate into various kinds of cells under different microenvironments or culture conditions. Besides, MSCs and fibroblasts demonstrate different gene expression patterns under subtle changes in oxygen concentrations and culture conditions. Therefore, it is challenging to distinguish MSCs from fibroblasts. However, our single-cell transcriptomic analysis demonstrates distinct differences between different subsets of MSCs and fibroblasts despite the intrinsic heterogeneity of gene expression, biological functions, and transcriptional regulation.

## Conclusion

Our study showed significant differences and similarities between 15 different cell subsets derived from HuMSCs, BMSCs, FSMSCs, and ADMSCs based on comprehensive analysis of integrated single-cell transcriptome data. Our study also demonstrated that several molecular markers were shared by distinct cell subsets and may be linked to their biological functions. Therefore, the single-cell transcriptome sequencing technique shows great promise in detecting the heterogeneity between cellular populations, classification of different cellular subsets, and elucidating the differences in biological functions between different subsets in a heterogenous population of cells. However, the single-cell technology is not perfect and needs further development. For example, it is limited for detecting low-expression genes that may have significant biological relevance. Moreover, it cannot postulate interactions between different cell subsets. Nevertheless, single-cell technology has enabled significant progress in cellular research and shows significant potential in broader clinical applications.

## Data Availability

Publicly available datasets were analyzed in this study. The data can be found here: BMSCs (GEO database: GSE115149, GSE162692); ADMSCs (GEO database: SRP148833); HuMSCs and FSMSCs (Mendeley database: https://data.mendeley.com/datasets/f4b2ykfv56/1. The treatment of two samples is detailed in an unpublished article. This unpublished article is another relevant work and submitted to another journal. Meanwhile, the copy of the unpublished article is available in Supplementary Material).

## Abbreviations

ADMSCs, adipose mesenchymal stromal cells; BMSCs, bone marrow mesenchymal stromal cells; FSMSCs, foreskin mesenchymal stromal cells; GEO, gene expression omnibus; HuMSCs, human umbilical cord mesenchymal stromal cells; ISCT, International Association for Cell Therapy; Log2FC, log2 fold change; MSCs, mesenchymal stromal cells; PCA, principal component analysis; PCs, principal components; RAS, regulon activity score; RSS, regulon-specific score; TSNE, *t*-distributed stochastic neighbor embedding; VIM, vimentin.
