# Supplementary figures and images for: Single-Cell Transcriptome Integration Analysis Reveals the Correlation Between Mesenchymal Stromal Cells and Fibroblasts
Source: Front Genet. 2022 Mar 7;13:798331. doi: 10.3389/fgene.2022.798331 (PMC8961367; doi:10.3389/fgene.2022.798331)

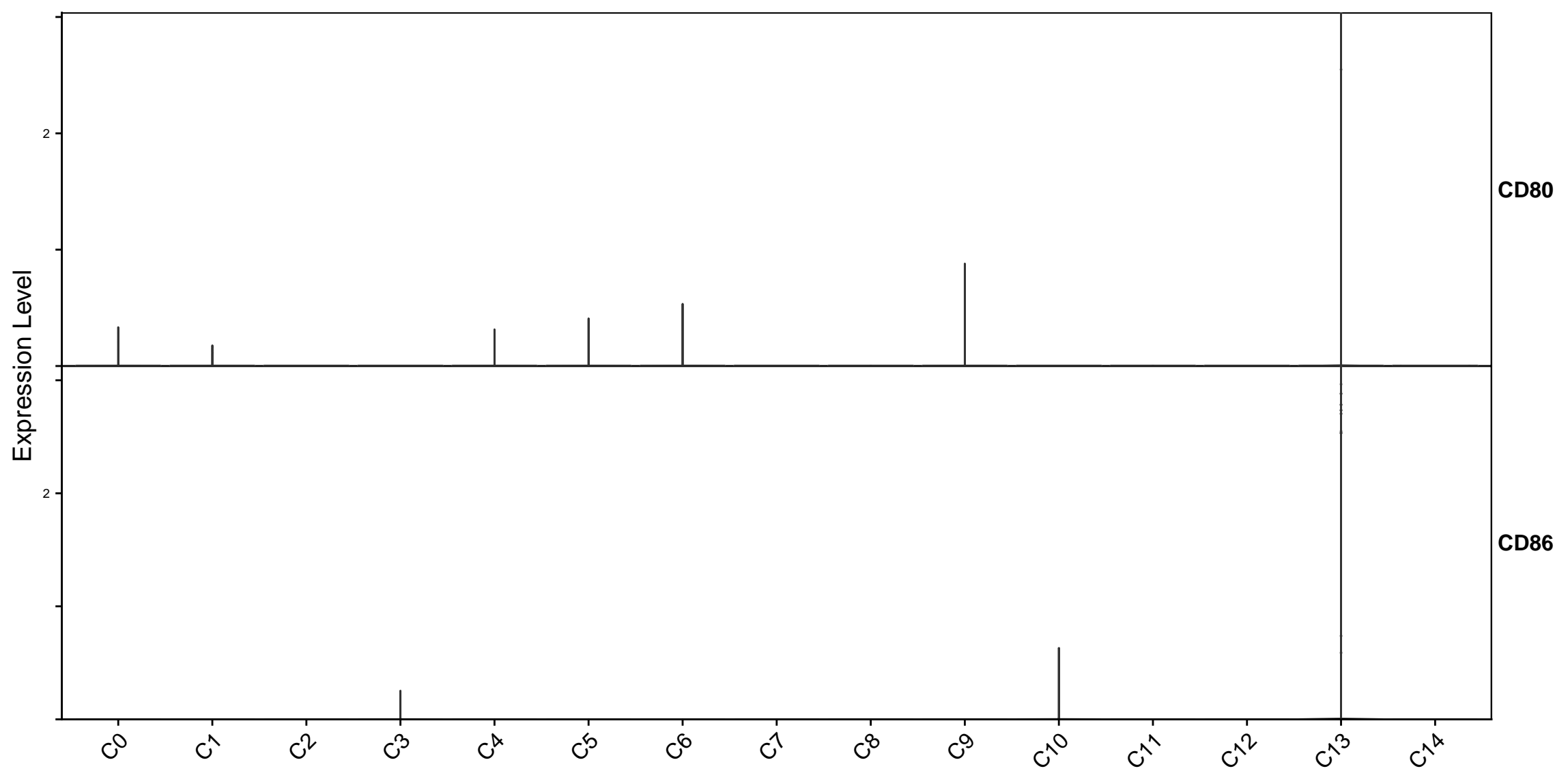

Supplement: Supplementary file 4 [file Image1.PDF]
